# A 14-year-old female with fever, rash, lymphadenopathy, and pancytopenia: a case report

**DOI:** 10.1186/s13256-019-2286-2

**Published:** 2020-01-26

**Authors:** Mojtaba Varshochi, Reyhaneh Ravanbakhsh Gavgani, Behrooz Naghili, Zhinus Bayatmakoo, Parinaz Poorshahverdi, Fatemeh Ravanbakhsh Gavgani

**Affiliations:** 1 Department of Infectious Disease, Faculty of Medicine, Tabriz University of Medical Sciences, Tabriz, 51666 Iran; 2 Department of Biological Sciences, School of Natural Sciences, University of Tabriz, Tabriz, Iran

**Keywords:** Fever, Rash, Lymphadenopathy, Pancytopenia

## Abstract

**Background:**

Anticonvulsant hypersensitivity syndrome is a rare adverse drug reaction associated with aromatic anticonvulsant drugs. This syndrome can range from mild cutaneous rash to drug reaction with eosinophilia and systemic symptoms that include fever, rash, lymphadenopathy, pancytopenia, and involvement of multiple internal organs. We aimed to report this case in the literature and make physicians aware of the uncommon symptoms of this syndrome when they prescribe antiepileptic medications in particular.

**Case presentation:**

A 14-year-old Middle Eastern female patient from Iran with free past medical and allergic history was admitted to hospital because of fever, rash, lymphadenopathy, and pancytopenia after taking anticonvulsants due to new-onset seizure. High fever and cutaneous rash along with lymphadenopathy following administration of anticonvulsant medications that could not be explained by other causes alerted the physician to the possibility of this syndrome. Our investigation revealed no further diagnosis and 1 week after discontinuation of the drugs, her symptoms were resolved. Anticonvulsant hypersensitivity syndrome is a diagnosis of exclusion and immediate discontinuation of the suspicious drugs is necessary. Hence, early recognition can prevent permanent multiorgan damage.

**Conclusions:**

Chlorpheniramine as a simple treatment was provided for this syndrome.

## Background

Anticonvulsant hypersensitivity syndrome (ACHS) is a rare but potentially fatal complication that is associated with aromatic antiepileptic drugs [1–4]. Women are more susceptible to this drug reaction, which indicates the role of sex hormones in the pathogenesis of ACHS [3, 5, 6].

Carbamazepine and phenytoin are the most common causes of ACHS, followed by lamotrigine [7].

## Case presentation

A 14-year-old Middle Eastern girl from Iran was admitted to Sina Medical Research and Training Hospital on 13 January 2019 because of fever and rash. She had been well until 4 weeks earlier, when she developed new-onset generalized tonic-clonic seizure with normal brain MRI and magnetic resonance venography (MRV).

An electroencephalogram (EEG) showed epileptic waves; thus, she was treated with phenobarbital and lamotrigine. Since then she had no more seizures. At time of admission (4 weeks after seizure treatment), she had fever and itchy skin rash, anorexia, nausea malaise, and fatigue of 1-week duration. She was otherwise fit and well with no significant past medical history of note and no history of familial disease.

On physical examination, she was alert, oriented, and conscious. She had multiple firm, non-tender, right and left cervical and inguinal lymph nodes as well as an erythematous maculopapular rash on her chest, abdomen, back, and upper limbs without palm and sole involvement that was highlighted with fever; the rash was inconspicuous when fever subsided.

Lungs, heart, abdomen, extremities, and neurologic examinations were normal. There was no hepatomegaly or splenomegaly.

Her vital signs on the first day of admission were: blood pressure, 105/65; body temperature, 38.7 axillary; pulse rate, 115; and respiratory rate, 20. Blood investigation immediately after admission revealed pancytopenia: white blood cells (WBC), 3140 cells/mm^3^; hemoglobin (Hb), 11.8 g/dl; and platelets (PLT), 118,000.

Cervical and abdominal ultrasounds were done and showed multiple lymph nodes in right posterior (5 × 23 mm), anterior (4 × 25 mm) cervical triangle, posterior occipital (6 × 15 mm), submandibular (5 × 14 mm) with echogenic hilum, left posterior (4 × 8, 4 × 11, 4 × 18 mm), anterior (2 × 11, 3 × 10 mm) cervical triangle with echogenic hilum, and submandibular (3 × 14 mm) without echogenic hilum with normal size of liver and spleen. A computed tomography (CT) scan of her chest (pulmonary and mediastinal) and abdomen was normal with bilateral inguinal lymphadenopathy. Cardiac echocardiography and electrocardiography (EKG) were both normal. Laboratory workup was summarized in Tables 1 and 2.

**Table 1:** Cell count with differentiation during hospitalization

	Day 1	Day 3	Day 4	Day 5	Day 6	Day 10	Day 14	Follow-up visit
White blood cells × 10^3^/μl	3.140	2.4	2.6	2.1	2.9	3.47	3.5	4.260
Neutrophils × 10^3^/μl	1.96					2.3		2.25
Lymphocytes × 10^3^/μl	1.04					0.72		1.41
Eosinophils × 10^3^/μl	0.50					0.17		0.21
Monocytes × 10^3^/μl	0.08					0.21		0.38
Basophils × 10^3^/μl	0.01					0.02		0.01
Hemoglobin g/dl	11.8	11	11.3	11.5	12.1	11.2	11	12
Platelets/mm^3^	118,000	102,000	108,000	93,000	103,000	205,000	231,000	267,000

**Table 2:** Laboratory tests during hospitalization

	Day 1	2 weeks before discharge	1 week before discharge	Discharge day	Follow-up visit
Aspartate aminotransferase	64 IU/l	91 IU/l	29 IU/l	19 IU/l	
Alanine aminotransferase	42 IU/l	71 IU/l	42 IU/l	30 IU/l	
Alkaline phosphatase	413 U/l	298 IU/l	252 IU/l	237 IU/l	
Total bilirubin	0.3 mg/dl				
Erythrocyte sedimentation rate	13 mm/hour		9 mm/hour		23 mm/hour
CRP	74 mg/dl		52 mg/dl		Negative
Lactate dehydrogenase	993 IU/l	1313 IU/l	1146 IU/l	674 IU/l	388 IU/l
Ferritin	700 ng/ml			115 ng/ml	
Triglyceride	135 mg/dl				
Creatinine	0.8 mg/dl				
Calcium	9.8				
Prothrombin time	12.4 seconds				
Partial thromboplastin time	32 seconds				
EBV, CMV, *Toxoplasma* IgM	Negative				
HIV Ab	Negative				
HBs Ag, HBc Ab, HCV Ab	Negative				
ANA, anti-dsDNA, P_ANCA, C_ANCA, anti-liver kidney microsomal antibodies, RF	Negative				
C3, C4, CH50	Normal				
Wright, Coombs Wright	Negative				
Rapid plasma reagin (RPR)	Negative				
Urine and blood culture	Negative				
Urine analysis	Normal				
PPD	Negative				
PBS	Normal				

*Ab* antibody, *ANA* antinuclear antibodies, *C_ANCA* cytoplasmic antineutrophil cytoplasmic antibody, *CMV* cytomegalovirus, *CRP* C-reactive protein, *dsDNA* double-stranded DNA, *EBV* Epstein–Barr virus, *HBc* hepatitis B core, *HBs Ag* hepatitis B surface antigen, *HCV* hepatitis C virus, *HIV* human immunodeficiency virus, *IgM* immunoglobulin M, *P_ANCA* perinuclear antineutrophil cytoplasmic antibody, *PBS* peripheral blood smear, *PPD* purified protein derivative, *RF* rheumatoid factor

Our first diagnosis was ACHS based on fever, rash, lymphadenopathy, and pancytopenia after taking anticonvulsants, so a neurology consult was done to change phenobarbital and lamotrigine to levetiracetam. Our differential diagnoses were viral infections, collagen vascular disease, Kikuchi-Fujimoto disease, and hematologic malignancy; all of which were ruled out (Tables 1 and 2). During her first week of hospitalization, our patient had daily intermittent fever with spikes in the mornings and at nights up to 39.5–40 °C which responded to parenteral acetaminophen. Furthermore, her lactate dehydrogenase (LDH) level increased, whereas WBC and PLT decreased. Laboratory evaluation revealed no further diagnosis.

Moreover, a peripheral blood smear (PBS), which was reported by an oncologist, was normal without malignancy. On the eighth hospital day, she underwent cervical lymph node excisional biopsy with respect to oncologist’s recommendation and she was given chlorpheniramine 4 mg every 12 hours after returning from the operating room. The next day, her fever and rash completely resolved and she got well.

A brief report of the lymph node biopsy by the pathologist was as follows: Two lymph node tissues with architectural distortion and depletion in germinal centers and diffuse infiltration of the histiocytes in the parenchyma and some mature lymphocytes. Two vague granuloma formations composed of epithelioid cells aggregate, surrounded by a rim of lymphocytes were noted. There were a few (scattered) large cells with vesicular nuclei and high nuclear cytoplasmic (N/C) ratio, which were more probably immunoblasts. There were also foci of necrosis and necrotic debris in the background. Therefore, immunohistochemistry (IHC) was recommended.

The IHC results for PAX5, CD5, CD30, CD68, and Ki-67 were not in favor of lymphoma. According to the pathologist’s point of view, necrotizing lymphadenitis was a possible diagnosis.

On the 16th hospital day, our patient was discharged while receiving levetiracetam and clonazepam. She was visited10 days after discharge. She had been in a good clinical condition without any problem or fever. Her latest laboratory investigation revealed: WBC, 4260 cells/mm^3^ (with normal eosinophil count as outlined in Table 1); Hb, 12 g/dl; PLT, 267,000; LDH, 388 IU/L; erythrocyte sedimentation rate (ESR), 23 mm/hour; and C-reactive protein (CRP), negative.

## Discussion and conclusions

ACHS, which is a rare but serious and potentially fatal complication, is associated with aromatic antiepileptic drugs, including phenytoin. ACHS occurs in 1 in 1000 to 1 in 10,000 patients treated with aromatic antiepileptic drugs such as carbamazepine, phenytoin, lamotrigine, oxcarbazepine, and phenobarbital, as well as allopurinol and the sulfonamides. This syndrome has a fatality rate of 10% [1–4].

Drug reaction to phenytoin was first recognized by Meritt and Putnam in the early 1930s. Then ACHS was described for the first time in 1950s [3, 4]. ACHS is a triad of fever, rash, and internal organ involvement [3]; cases are often underreported and unrecognized [1, 3].

Since women, especially fertile women, are more susceptible to this drug reaction, female sex hormones might have a role in the pathogenesis of ACHS [3, 5]. This tendency has previously been reported for lamotrigine [6].

ACHS has an autosomal dominant inheritance pattern. It is a type IV T cell-mediated delayed hypersensitivity reaction with high susceptibility between siblings and other first-degree relatives of affected patients [2, 3].

Studies showed that carbamazepine and phenytoin are the most common causes of ACHS, and lamotrigine is the next most common etiologic agent [7]. A high rate of cross-reactivity among aromatic antiepileptic drugs is of concern and it might be as high as 80% [4].

Factors which have been associated with ACHS are the human leukocyte antigen HLA-A*3101, HLA-B*1502, human herpesvirus 6 (HHV6), human herpesvirus 7 (HHV7), cytomegalovirus (CMV), and Epstein–Barr virus (EBV) [1, 3, 8–11].

Drug reaction with eosinophilia and systemic symptoms (DRESS) or drug-induced hypersensitivity syndrome (DIHS) are used as equivalent to ACHS, that is, a severe idiosyncratic reaction characterized by various features but symptoms classically include fever, rash, lymphadenopathy, pharyngitis which is common, and possibly other organ involvement [1–4, 7]. When severe, the syndrome can include hepatitis, megaloblastic anemia, rhabdomyolysis, and arteritis.

In most patients, the reaction begins 2 to 6 weeks after the initiation of the mentioned medications [1–3]. Early diagnosis is vital because of the high mortality rate [2, 12], and early recognition can prevent permanent multiorgan damage [2]. Diagnosis of ACHS is based on a history of antiepileptic drugs exposure and clinical symptoms. Signs and symptoms usually occur within 3 months of initiating treatment with an anticonvulsant, most often within 2 to 6 weeks [2]. When ACHS is suspicious due to signs and symptoms that occur typically 2–6 weeks after the initiation of antiepileptic drugs, discontinuation of them is essential [2, 4].

The optimal treatment approach is controversial [12]. Treatments include antihistamines (H1-receptor blockers), epinephrine, glucocorticoids, anabolic steroids, anti-gonadotropic agents, and airway management, depending on the severity of the condition [2, 4, 12]. N-acetylcysteine may be efficacious in hepatitis [13]. Some studies reported severe diseases and prolonged hospital stay for skin drug-related complications [12]. In patients who were treated with intravenous immunoglobulin and systemic corticosteroids, the length of hospitalization was short and all recovered without complication [12, 14].

Complications of DRESS based on age can be divided into two major types: in young patients, autoimmune diseases, specifically Graves’ disease, type 1 diabetes mellitus, and autoimmune hemolytic anemia may occur prominently; older patients are more susceptible to end-organ failure such as chronic renal failure [15].

The most probable causes of lymphadenopathy and pancytopenia according to our patient history and physical examination were drug reaction, lymphoproliferative disorders, brucellosis, human immunodeficiency virus (HIV), EBV, CMV, hepatitis B, hepatitis C, parvovirus B19, and rheumatologic diseases, especially systemic lupus erythematosus (SLE); all of which were ruled out based on the laboratory workup. Because of absence of severe anemia and arthralgia, parvovirus B19 was excluded. Toxoplasmosis causes fever and lymphadenopathy but its serology was negative in our patient.

ACHS was our first probable diagnosis because our patient initially had a seizure without other systemic symptoms, she was treated with antiepileptic drugs and 3 weeks later she presented with fever, rash, lymphadenopathy, and pancytopenia. At her first complete blood cell count, no eosinophilia and/or monocytosis and/or atypical lymphocytosis were detected, which is a major component of antiepileptic hypersensitivity syndrome (AHS)/DRESS syndrome. Another diagnosis was rapidly progressive lymphoproliferative disease but PBS and ESR were normal, although it could not be completely ruled out.

According to symptoms, age, sex and Asian ethnicity, Kikuchi-Fujimoto disease was considered. Kikuchi-Fujimoto disease is a rare histiocytic necrotizing lymphadenitis with a benign course and unknown etiology; it is characterized by cervical lymphadenopathy and fever. Histopathology of the involved lymph nodes differentiated it from other diseases that it mimics. In this disease, a complete blood cell count is often normal, although anemia, leukopenia, thrombocytopenia, and pancytopenia have been reported. High-level LDH and normal or elevated ESR are other nonspecific findings.

Our patient initially had a seizure and 3 weeks after initiation of phenobarbital and lamotrigine her systemic symptoms began; after discontinuation of all drugs and administration of chlorpheniramine 4 mg for two doses her symptoms were completely resolved.

Pathological study ruled out malignant etiology, so a diagnosis of ACHS was confirmed. Our patient was then discharged. She had no problems at the following visit.

According to the reports of autoimmune diseases subsequent to ACHS, our patient would be followed-up with thyroid function tests and the possibility of diabetes mellitus in the future.

Since genetic factors may predispose individuals to ACHS, it may occur in our patient’s siblings after taking anticonvulsants. Therefore, her parents became aware of this issue. Given that her history of seizure had remained a dilemma, it was better to follow-up for rheumatologic diseases, especially SLE.

In conclusion, ACHS is a rare genetic disease, which may occur after taking anticonvulsant drugs. Physicians should be aware of various symptoms of this syndrome to recognize it in patients with fever, leukopenia, rash, and lymphadenopathy after taking these drugs. Accordingly, they should pursue a quick therapeutic action, including discontinuing medications and prescribing antihistamines.

## Data Availability

All data and materials related to this report are accessible at any time upon request.

## Abbreviations

ACHS: Anticonvulsant hypersensitivity syndrome
CMV: Cytomegalovirus
DRESS: Drug reaction with eosinophilia and systemic symptoms
EBV: Epstein–Barr virus
ESR: Erythrocyte sedimentation rate
Hb: Hemoglobin
IHC: Immunohistochemistry
PBS: Peripheral blood smear
PLT: Platelets
SLE: Systemic lupus erythematosus
WBC: White blood cells
